# Health Disparities in Presentation, Treatment, Genomic Testing, and Outcomes of Pancreatic Cancer in Hispanic and Non-Hispanic Patients

**DOI:** 10.1007/s40615-022-01486-1

**Published:** 2023-04-18

**Authors:** Ritika Halder, Sumana Veeravelli, Ce Cheng, Ricardo J. Estrada-Mendizabal, Alejandro Recio-Boiles

**Affiliations:** 1 University of Arizona Internal Medicine Residency Program, Tucson, AZ USA; 2 Tecnológico de Monterrey Escuela de Medicina y Ciencias de la Salud, Monterrey, N.L. Mexico; 3 University of Arizona Cancer Center, Tucson, AZ USA

**Keywords:** Hispanic, Pancreatic, Adenocarcinoma, Disparities

## Abstract

**Background:**

There are few conflicting results regarding the treatment and outcomes of Hispanic patients with pancreatic cancer. This study comprehensively evaluated the differences in baseline characteristics, treatments, genomic testing, and outcomes among Hispanic (H) and Non-Hispanic (NH) patients with early-stage (ES) and late-stage (LS) pancreatic cancer (PC).

**Methods:**

This is a retrospective analysis from 2013 to 2020 of 294 patients with pancreatic ductal adenocarcinoma; data collected included patient demographics, clinical characteristics, treatment regimens, response, germline and somatic genetic testing, and survival outcomes. Excluded those with insufficient data. Univariate comparisons used parametric and nonparametric tests as appropriate to evaluate for differences between H and NH groups. Fisher’s exact tests were performed to evaluate the difference in frequency. Kaplan–Meier and Cox regression analysis assessed the survival.

**Results:**

The analysis included 198 patients who had a late-stage disease and 96 patients with early-stage disease at the time of diagnosis. Among the early-stage patients, the median age at diagnosis was 60.7 years in the H versus 66.7 years in the NH (*p* = 0.03). No other differences were observed in baseline characteristics, treatments offered, and median overall survival (NH 25 vs. H 17.7 months, *p* = 0.28). Performance status, negative surgical margins, and adjuvant therapy were clinically significant and univariable with improved OS (*p* < 0.05), regardless of ethnicity. Hispanic patients with early pancreatic cancer were noted to be at a greater risk of death with a statistically significant hazard ratio of 3.1 (*p* = 0.005, 95% CI, 1.39–6.90). Among the late-stage patients, Hispanic patients with ≥ 3 predisposing risk factors for pancreatic cancer were 44% vs. 25% of NH (*p* = 0.006). No significant differences were noted in baseline characteristic treatments, progression-free, and median overall survivals (NH 10.0 vs. 9.2 months, *p* = 0.4577). In the late-stage genomic testing, germline testing performed in NH 69.4% vs. H 43.9% (*p* = 0.003) revealed no difference among groups. For the somatic testing, the pathogenic variants with actionable mutations were 2.5% of NH and 17.6% of H patients (*p* = 0.03).

**Conclusion:**

Hispanic patients with early-stage pancreatic adenocarcinoma present at a younger age and with more risk factors in the late stage. These patients have significantly lower overall survival compared to their non-Hispanic counterparts. Hispanic patients in our study were 2.9 less likely to receive germline screening and more like to have somatic genetic actionable pathogenic variants. Overall, only a minority of all patients were enrolled in a pancreatic cancer clinical trial or offered genomic testing, highlighting a critical need and missed opportunity in advancing progress and improving outcomes for this disease, mainly in the underrepresented Hispanic population.

## Introduction

Pancreatic cancer is the 10th most common cause of cancer in the USA and the 3rd leading cause of cancer-related death and 2nd among gastrointestinal malignancies [1]. The American Cancer Society estimates that in 2021, approximately 62,210 new cases of pancreatic cancer will be diagnosed in the USA and 49,830 people will die from the disease [1]. Although the incidence of pancreatic cancer has been stable over the last decade, death rates have been climbing an average of 0.3% each year from 2008 to 2017 [2]. The treatment of pancreatic cancer requires a multidisciplinary approach with surgical resection as the only potentially curative option. The majority (53%) of patients are diagnosed at an advanced stage and face a 5-year survival rate of 3.1% [1]. Even the minority of patients who are candidates for resection face a poor prognosis with a 5-year survival rate of 30% [3]. Systemic therapy remains a mainstay of treatment in the adjuvant resectable and unresectable/metastatic settings [4].

Clinical trials in pancreatic cancer have historically underrepresented Hispanic patients, particularly noted in the landmark phase III trials showing efficacy in pancreatic cancer—the PRODIGE 4/ACCORD 11 trial which studied a mainly French-only population in which ethnic background was not reported [5], the MPACT 2013 trial in which baseline characteristics identified only 6% of the study population as Hispanic [6], and the POLO 2019 trial in which there was multinational recruitment and Hispanics represented 3% of all the patients [7].

Unfortunately, racial disparities play a large part in nearly every aspect of pancreatic cancer care in the USA, with well-studied inequalities documented particularly among Blacks [8]. Disparities have been identified in treatments, with lower rates of radiation [9] and overall lower utilization of multimodality therapy among Hispanics [10], particularly among those presenting at an early stage [11]. Quality of life measures have also been demonstrated to be lower among Hispanics [12]. Hispanic patients were found to be less likely to travel to an academic program for treatment, which correlated to poorer perioperative outcomes and overall survival [13]. However, other studies have demonstrated no disparities in treatment or outcomes [14], some studies even suggesting survival benefits among Hispanics. In one CA population-based study among patients with unresectable pancreatic cancer, Hispanics were significantly more likely to reach the 5-year survival milestone as compared to non-Hispanics [15].

Previous retrospective reviews have demonstrated conflicting results regarding the treatment and outcomes of Hispanic patients with pancreatic cancer. Because cancer incidence and mortality vary across racial/ethnic groups and geographic regions in the USA [1], our group investigated the health disparities among patients with pancreatic cancer at an NCI-designated comprehensive cancer center uniquely located in AZ with the largest minority group being the Hispanic population, predominantly Mexican Americans, historically underrepresented in clinical research.

## Materials and Methods

This is a retrospective analysis of 198 patients with advanced-stage pancreatic cancer treated at the University of Arizona Cancer Center from 2013 to 2020, comprising 41 Hispanic and 157 non-Hispanic patients. We also included 96 patients with early-stage pancreatic cancer, of whom 15 were Hispanic and 81 were non-Hispanic. Early-stage pancreatic cancer patients were defined as an upfront resectable or borderline resectable disease that had a surgical procedure with/without peri-operative therapies. Late-stage pancreatic cancer patients were classified as those who progressed to or presented with unresectable and/or metastatic disease and proceeded with at least one line of systemic therapy. Patients with insufficient documentation for data collection were excluded. Data were collected on patients’ demographics, clinical characteristics, treatment regimens, responses, germline and somatic genetic testing, and survival outcomes. Pancreatic risk factors considered were cigarette smoking, alcohol consumption, obesity, diabetes mellitus, chronic pancreatitis, pancreatic cysts, and personal and family history of genomic syndromes. Univariate comparisons used parametric ANOVA and nonparametric Kruskal–Wallis rank sum tests as appropriate to evaluate for differences between Hispanic and non-Hispanic groups. Fisher’s exact tests were performed to evaluate the difference in frequency of each gene variable. Kaplan–Meier and Cox regression analyses were used to evaluate overall survival (OS) and progression-free survival (PFS), while comparisons across ethnicity used log rank tests. Statistical analyses were performed using Stata16 (Stata Corp, College Station, TX, USA).

## Results

### Early-Stage Pancreatic Cancer

Baseline characteristics between Hispanics (H) and NH (non-Hispanics) patients, including sex, tumor location, and median CA 19–9 levels, in the early-stage pancreatic cancer population were balanced. There was a statistically significant difference in the median age at diagnosis—60.7 years in the H group versus 66.7 years in the NH group (*p* = 0.037). Otherwise, both groups had no other significant difference in baseline characteristics, with the following averages: 54% male, performance status ≥ 80 [ECOG 0–1] of 88%, tumor location with 77% in the head of the pancreas, and median CA 19–9 level 124 U/ml [Table 1]. Treatments offered to each group did not greatly differ with 18% receiving neoadjuvant chemotherapy, 59% adjuvant chemotherapy, 28% radiation, and 12% enrolled in a clinical trial. Of note, in this population, 73% of Hispanics had complete resection (R0, negative margins) after surgery, compared to only 41% of non-Hispanics (*p* = 0.026) [Table 2].

**Table 1:** Baseline characteristics in early-stage pancreatic cancer patients

	Non-Hispanic	Hispanic	Total	*p*-value
	*N* = 81 (84.4%)	*N* = 15 (15.6%)	*N* = 96	
Age (in years)	66.7 (10)	60.7 (9.6)	65.8 (10.2)	0.0037
Sex				0.62
Female	38 (47%)	6 (40%)	44 (46%)	
Male	43 (53%)	9 (60%)	52 (54%)	
Race				0.073
White	70 (86%)	11 (73%)	81 (84%)	
Black	2 (2%)	0 (0%)	2 (2%)	
Native American	4 (5%)	0 (0%)	4 (4%)	
Asian	2 (2%)	0 (0%)	2 (2%)	
Unknown	3 (4%)	4 (27%)	7 (27%)	
Karnofsky scale				0.79
70	10 (12%)	2 (13%)	12 (13%)	
80	14 (17%)	4 (27%)	18 (19%)	
90	35 (43%)	6 (40%)	41 (43%)	
100	22 (27%)	3 (20%)	25 (26%)	
Carbohydrate antigen 19–9 median (Q1, Q3)	123 (24.0, 547)	177 (25, 1727)	124 (24.5, 591)	0.50
Tumor location				0.94
Head	62 (77%)	12 (80%)	74 (77%)	
Body	8 (10%)	1 (7%)	9 (9%)	
Tail	7 (9%)	2 (13%)	9 (9%)	
Overlapping	4 (5%)	0 (0%)	4 (4%)

**Table 2:** Treatments in early-stage pancreatic cancer patients

	Non-Hispanic	Hispanic	Total	*p*-value
	*N* = 81 (84.4%)	*N* = 15 (15.6%)	*N* = 96	
Surgical resection margins				0.026
Positive (R1)	46 (59%)	4 (27%)	50 (54%)	
Negative (R0)	32 (41%)	11 (73%)	43 (46%)	
Neoadjuvant	13 (16%)	4 (27%)	15 (16%)	0.32
Adjuvant	49 (60%)	8 (53%)	57 (59%)	0.60
Clinical trial	10 (13%)	1 (7%)	11 (12%)	1.00
Radiation	22 (28%)	4 (27%)	26 (28%)	1.00

**Fig. 1:**
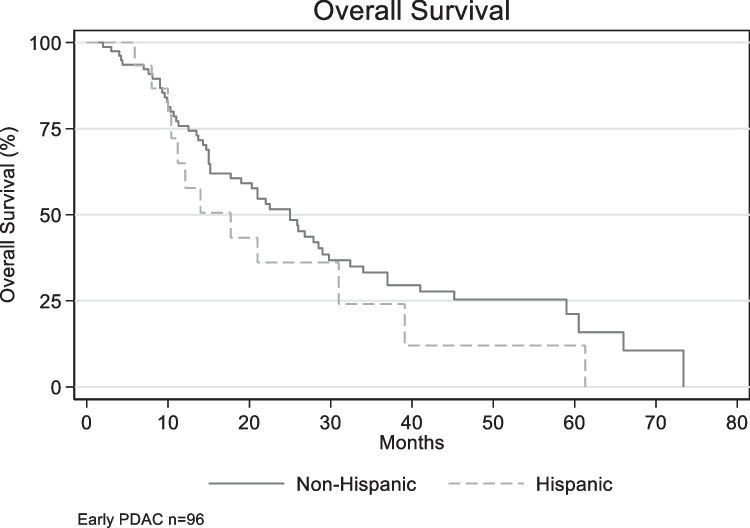
Overall survival by ethnicity in early-stage pancreatic cancer

**Fig. 2:**
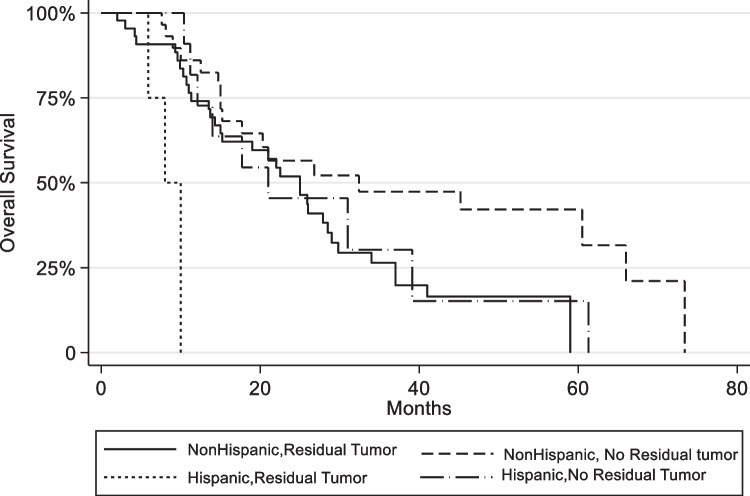
Survival by ethnicity and resection margin

Among early-stage pancreatic cancer patients, there was no difference in median overall survival (mOS) between NH with mOS of 25 months, and H with mOs of 17.7 months (*p* = 0.2814) [Fig. 1]. Performance status ≥ 90, negative surgical margins, and adjuvant therapy were clinically significant univariable with improved OS (*p* < 0.05). In a model controlling for age, surgical margins, performance status > 80, radiation, and systemic therapy (adjuvant vs. neoadjuvant vs. both), Hispanics were at a greater risk of death with a statistically significant hazard ratio of 3.1 (*p* = 0.005, 95% CI, 1.39–6.90) [Fig. 2].

### Late-Stage Pancreatic Cancer

Baseline characteristics in the advanced and metastatic stage pancreatic cancer population were also balanced between H and NH patients including median age at diagnosis of 66, 58% male, 58% performance status ≥ 80 [ECOG 0–1], tumor location with 61% in the head of the pancreas, 41% requiring a biliary stent, and median CA19-9 level 406 U/ml. Among late-stage patients, Hispanics had a significantly higher proportion of patients with ≥ 3 predisposing risk factors for pancreatic cancer, with about 44% of H with risk factors vs. 25% of NH with risk factors (*p* = 0.0066) [Table 3].

**Table 3:** Baseline characteristics in late-stage pancreatic cancer patients

	Non-Hispanic	Hispanic	Total	*p*-value
	*N* = 157 (79.3%)	*N* = 41 (20.7%)	*N* = 198	
Age (in years)	66.8 (10.8)	64 (10.1)	66.2 (10.7)	0.1237
Sex				0.9470
Female	66 (42%)	17 (41.5%)	83 (41.9%)	
Male	91 (58%)	24 (58.5%)	115 (58.1%)	
Race				< 0.001
White	140 (89.2%)	26 (63.4%)	166 (83.8%)	
Black	4 (2.5%)	1 (2.4%)	5 (2.5%)	
Native American	7 (4.5%)	0 (0%)	7 (3.5%)	
Asian	3 (1.9%)	0 (0%)	3 (1.5%)	
Pacific Islander	1 (0.6%)	0 (0%)	1 (0.5%)	
Unknown	2 (1.3%)	14 (34.1%)	16 (8.1%)	
Karnofsky scale				0.9123
70	26 (22.6%)	9 (24.3%)	35 (23.0%)	
80	10 (8.7%)	4 (10.8%)	14 (9.2%)	
90	42 (36.5%)	12 (32.4%)	54 (35.5%)	
100	15 (13%)	5 (13.5%%	20 (13.2%)	
Carbohydrate antigen 19–9 median (Q1, Q3)	466 (106, 2668)	263 (34, 2595)	406 (69.5, 2631.5)	0.4256
Total bilirubin median (Q1, Q3)	1 (0.5, 9)	2.5 (0.6, 9.5)	1.0 (0.6, 9.0)	0.7374
Tumor location				0.2644
Head	96 (61.1%)	25 (61.0%)	121 (61.1%)	
Body	26 (16.6%)	3 (7.3%)	29 (14.6%)	
Tail	14 (8.9%)	7 (17.1%)	21 (10.6%)	
Overlapping	21 (13.4%)	6 (14.6%)	27 (13.6%)	
No of metastatic sites				0.5849
1	124 (79.0%)	33 (80.5%)	157 (79.3%)	
2	29 (18.5%)	8 (19.5%)	37 (18.7%)	
3	4 (2.5%)	0 (0%)	4 (2.0%)	
Number of risk factors				0.0097
0	32 (20.4%)	1 (2.4%)	33 (16.7%)	
1	26 (16.6%)	8 (19.5%)	34 (17.2%)	
2	59 (37.6%)	14 (34.1%)	73 (36.9%)	
3	30 (19.1%)	10 (24.4%)	40 (20.2%)	
4	10 (6.4%)	7 (17.1%)	17 (8.6%)	
5	0 (0%)	1 (2.4%)	1 (0.5%)	
Categories of risk factors				0.0066
0	32 (20.4%)	1 (2.4%)	33 (16.7%)	
1–2	85 (54.1%)	22 (53.7%)	107 (54.0%)	
3 >	40 (25.5%)	18 (43.9%)	58 (29.3%)	

In total, 9%, 33%, and 16% of patients received neoadjuvant therapy, surgery, and adjuvant therapy, respectively. Multimodality therapy including chemoradiotherapy (13%), ≥ 2 lines of systemic therapy (21%), and clinical trial enrollment (8%) was balanced among these groups [Table 4]. First-line systemic therapy selection between groups demonstrated no significant difference between FOLFIRINOX (N5 H and N23 NH), and gemcitabine/abraxane (N11 H and N40 NH) (*p* = 1).

**Table 4:** Treatments in late-stage pancreatic cancer patients

	Non-Hispanic	Hispanic	Total	*p*-value
	*N* = 157 (79.3%)	*N* = 41 (20.7%)	*N* = 198	
Surgery	57 (36.3%)	9 (22%)	66 (33.3%)	0.0825
Neoadjuvant chemotherapy	16 (10.2%)	2 (4.9%)	18 (9.1%)	0.2920
Adjuvant chemotherapy	26 (16.6%)	6 (14.6%)	32 (16.2%)	0.7654
First Line	77 (49%)	26 (63.4%)	103 (52%)	0.1010
Second line	33 (21%)	9 (22%)	42 (21.2%)	0.8966
Third line	6 (3.8%)	4 (9.8%)	10 (5.1%)	0.1250
Clinical trial	13 (8.3%)	3 (7.3%)	16 (8.1%)	0.8403
Two modalities	24 (15.3%)	2 (4.9%)	26 (13.1%)	0.0789
Three modalities	21 (13.4%)	2 (4.9%)	23 (11.6%)	0.1305

**Fig. 3:**
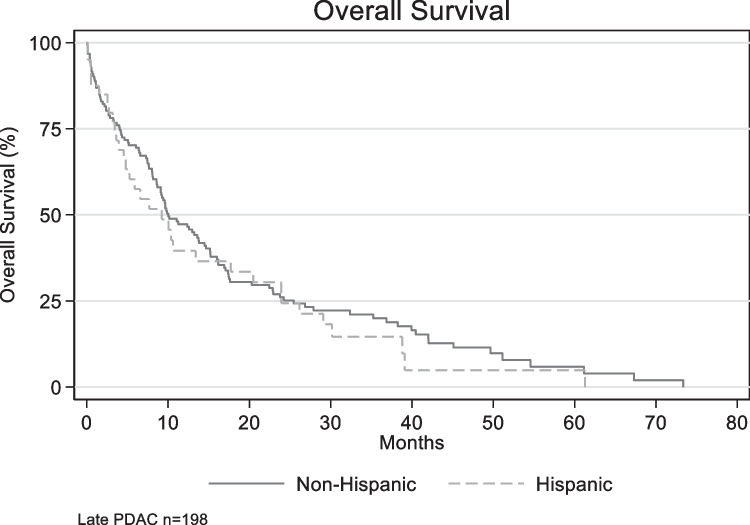
Overall survival by ethnicity in late-stage pancreatic cancer

Among late-stage pancreatic cancer patients, mOS was 9.2 months (95% CI 4.6–17.7) and 10.0 months (95%CI 8.7–13.8) for H vs. NH (*p* = 0.4577) [Fig. 3]. mPFS was 5.9 months and 8.6 months for H vs. NH (*p* = 0.33). A Cox regression model controlling for cofounders found no difference in OS (HR 1.2, 95% CI 0.7–1.9, *p* = 0.54) or PFS (HR 1.1, 95% CI 0.7–1.6, *p* = 0.70). FOLFIRINOX vs. gemcitabine/abraxane (all patients) in the front-line provided the best OS and no difference among them, 17.5 vs. 17.49 months (*p* = 0.95).

Sixty-four percent and 49% of the late-stage pancreatic cancer patients had germline and somatic genomic testing, respectively. In the germline mutation testing group, 69.4% non-Hispanic and 43.9% Hispanic patients were tested (*p* = 0.003). There was no statistically significant difference among the germline genomic testing of pathogenic variants (BRCA1/2, HRR deficiency, tissue-agnostic, and/or inherited cancer syndrome mutations), variants of unknown significance, negative testing results, or patients who declined to test/were not tested. In the somatic genomic testing group, the pathogenic variants of actionable mutations showed a statistically significant difference between non-Hispanic (2.5%) and Hispanic (17.6%) patients (*p* = 0.0364). Additionally, PD1/L1 IHC positive was not different between groups. Among the most common mutations by the number of variations were KRAS (74.2%), TP53 (55.6%), CDKN2A (19.5%), SMAD4 (16.45), and ATM (8.2%), with no statistically significant differences between groups [Table 5].

**Table 5:** Germline and somatic genetic testing

Late stage	Non-Hispanic	Hispanic	Total	*p*-value
	*N* = 157 (79.3%)	*N* = 41 (20.7%)	*N* = 198	
*Germline test N (%)*	*109 (69.4)*	*18 (43.9)*	*127 (64.1)*	**0.003335**
Pathogenic variant (*)	22 (20.1)	3 (16.6)	25 (19.6)	1
*BRCA1/2	8 (7.3)	1 (5.5)	9 (7.0)	1
*HRR deficiency	14 (12.8)	1 (5.5)	12 (9.4)	0.6934
*Tissue-agnostic	1 (0.9)	1 (5.5)	2 (1.5)	0.2643
*Inherited cancer syndrome	14 (12.8)	3 (16.6)	17 (13.3)	0.7087
Variants of unknown significance (#)	27 (24.7)	7 (38.8)	34 (26.7)	0.252
Negative testing	45 (41.2)	10 (24.3)	55 (43.3)	0.3088
Decline/not test	13 (11.9)	5 (12.1)	18 (14.1)	0.1349
*Somatic test N (%)*	*80 (50.9)*	*17 (41.4)*	*97 (48.9)*	0.2977
Pathogenic variant (*)				
*BRCA1/2	2 (2.5)	0 (0)	2 (2.0)	1
*HRR deficiency	11 (13.7)	3 (17.6)	14 (14.4)	0.707
*Tissue-agnostic	0 (0)	1 (5.8)	1 (1.0)	0.1753
*Actionable mutations	2 (2.5)	3 (17.6)	5 (5.1)	**0.0364"**
PD1/L1 positive	14 (17.5)	4 (9.7)	18 (18.5)	0.513
Most common mutations by variations				
KRAS	62 (77.5)	10 (58.8)	72 (74.2)	0.1313
TP53	47 (58.7)	7 (41.1)	54 (55.6)	0.2821
CDKN2A	16 (20.0)	3 (17.6)	19 (19.5)	1
SMAD4	14 (17.5)	2 (11.7)	16 (16.4)	0.7298
ATM	7 (8.7)	1 (5.8)	8 (8.2)	1

Targeted therapy FDA approval olaparib BRCA1/2. Homologous recombination-related may Include BRCA1/2, PALB2, ATM, BAP1, BARD1, BLM, BRIP1, CHEK2, FAM175A, FANCA, FANCC, NBN, RAD50, RAD51, RAD51C, and RTEL1. Tissue-agnostic therapy FDA approval dMMR, MLH1, MSH2, MSH6, PMS2, MSI-H, TMB-high, NTRK, BRAF. Actionable somatic mutations FDA off-label, including but not limited to, mutations (“NH-KRAS G12C and CDK6, Hispanic- CDK6, HER2[2]”), no fusions. Associated Hereditary risks may include BRCA1/2, PALB2, ATM, CDKN2A, STK 11, APC, dMMR, PRSS1, SPINK1, CFTR, and P53

Bolded entries are statistically significant *p*-values

## Discussion

To our knowledge, our group presents the largest and most comprehensive review of Hispanic pancreatic cancer from early to late-stage. Our review compares Hispanic and non-Hispanic patient group baseline characteristics at presentation, outcomes, and analysis of germline to somatic genetic testing published up to date. While the Hispanic population with pancreatic adenocarcinoma presented at a younger age in the early stage and with more risk factors in the late stage, there was no difference in the number or modality of treatments offered at our Comprehensive Cancer Center. Notably, only one Hispanic subgroup analysis showed a significantly lower overall survival compared to their non-Hispanic counterparts—those with positive surgical resection margins (R1, HR 3.1, *p* = 0.005), despite having higher rates of negative margins compared (R0, *p* = 0.026) [Fig. 2]. Furthermore, our cancer center’s experience treating a large Hispanic population showed OS and PFS outcomes comparable to historic phase III trials in early [16] and late-stage [6, 17] pancreatic cancer with no significant difference between ethnic groups.

Patients with early-stage pancreatic cancer with good performance status, a negative surgical margin, and adjuvant chemotherapy demonstrated an improvement in the OS. These results were expected and concur with the current literature, trial outcomes, and guideline recommendations [18, 19]. On the other hand, current evidence showing a benefit of neoadjuvant therapy in these patients remains controversial, we continue to recommend a case-by-case discussion on the selection of this approach. Regarding the late-stage cohort, no differences were seen between first-line systemic chemotherapy options between FOLFIRINOX and gemcitabine/abraxane, and both showed the best OS, both being category 1 recommendations [19].

Among Hispanic patients with metastatic pancreatic cancer, the prevalence of germline BRCA1 and 2 entries into the POLO trial was 7.7% (N65 patients, BRCA1 3.1%, and BRCA2 4.6%) [20]; this did not significantly differ from the NH patients (7.3%, N2061 patients, BRCA1 2.2%, BRCA2 5.1%). Our data has similar results, noting the prevalence of BRCA 1 and 2 in the Hispanic population at 5.5% (N18 patients, BRCA1 0%, BRCA2 5.5%) and the non-Hispanic population at 7.3% (N109, BRCA1 0%, BRCA2 7.3%). This is likely because that Hispanic breast cancer patients have a four- to fivefold lower likelihood of being screened for germline BRCA mutations than non-Hispanic patients, as described by Dean et al. Compared to non-Hispanic patients in our Hispanic patients were 2.9 less likely to receive germline screening (95% CI, 1.435 to 5.867, *p* = 0.003) (21). Although our review revealed an increasing germline (64%) and somatic (48%) genetic testing performed, these also suggest an opportunity to advance efforts in genomic sequencing recommended by ASCO [21].

Only 4.6% of patients with pancreatic cancer are enrolled in a pancreatic cancer trial [22], which highlights a critical need and missed opportunity in advancing progress and improving outcomes for this disease. In our retrospective study, we identified that approximately 12% of early stage and 8% of late stage of all Hispanic and non-Hispanic patients were enrolled in a clinical trial, still below the goal of 20% enrollment in clinical trials by the ASCO recommendations. An increasing number of therapies are emerging to treat specific genomic cancer biomarkers and could increase the enrollment of patients in clinical trials to advance knowledge and improve outcomes for this dismal disease [23]. Furthermore, it presents an opportunity to reduce the healthcare disparities and analyze a more diverse patient base in future RCTs for pancreatic cancer.

Unfortunately, less than 2% of late-stage-cancer pancreatic cancer will have FDA-approved tissue-agnostic immunotherapy indications, noted in our review of germline and somatic testing with an N3 (1.5%) of this very rare group, to divide and perform appropriate analysis. Furthermore, clinical trial participation at our cancer center may have included immunotherapy, but due to the small number of patients and multiple potential trial treatment interventions, it was not considered reasonable to focus on each modality including immunotherapeutics, to prove a disparity to access.

Tables 1 and 3 have a clear breakdown of race and ethnicity as directed to be collected by the NHI/NCI/census providing the groups used in the diversity calculations. We acknowledge the limitation of the missing data when collecting the country of origin. To my knowledge, there are no other ethnicities unless referring to the ancestral or country of origin.

We recognize there is an inherited selection bias in a retrospective study of patients presenting at a single institution that a future clinical trial design and/or inter-institutional collaborative studies could address by focusing on the ethnicity differences among patients with pancreatic cancer. Our study is limited by this bias in addition to the small sample size. This study includes patients presenting to a single cancer center and recognizes that standards of practices may vary between hospitals. Though there is a lack of statistical power due to lower population size, our study is still able to make comparisons between different groups. This was an exploratory study; however, thus multiplicity is a study limitation. Due to the multiple subgroup comparisons and analysis of multiple outcomes, the likelihood that an association by chance could have been deemed causal or significant is present.

## Data Availability

The data that support the findings of this study are available from the corresponding author R. H., upon reasonable request.
